# Substitution Effects of NaCl by KCl and CaCl_2_ on Lipolysis of Salted Meat

**DOI:** 10.3390/foods8120595

**Published:** 2019-11-20

**Authors:** Fabiane M. Nachtigall, Vitor A. S. Vidal, Radha D. Pyarasani, Rubén Domínguez, José M. Lorenzo, Marise A. R. Pollonio, Leonardo S. Santos

**Affiliations:** 1 Intituto de Ciencias Químicas Aplicadas, Universidad Autónoma de Chile, Talca 3467987, Chile; fabiane.manke@uautonoma.cl; 2 Faculdade de Engenharia de Alimentos, Universidade Estadual de Campinas, Campinas 13083-862, São Paulo, Brazil; vitorandrevidal@gmail.com; 3 Vicerrectoría de Investigación y Postgrado, Universidad Católica del Maule, Talca 3460000, Chile; radhakeerthi@gmail.com; 4 Centro Tecnológico de la Carne de Galicia, Parque Tecnológico de Galicia, 32900 San Cibrao das Viñas, Galicia, Ourense, Spain; rubendominguez@ceteca.net (R.D.); jmlorenzo@ceteca.net (J.M.L.); 5 Instituto de Química de Recursos Naturales, Universidad de Talca, Talca 3462227, Chile

**Keywords:** salted meat, salt substitutes, lipolysis, lipid oxidation, fatty acids, mass spectrometry

## Abstract

The objective of this study was to investigate the reduction and partial substitution effects of sodium chloride (NaCl) by potassium chloride (KCl) and calcium chloride (CaCl_2_) on lipolysis and lipid oxidation in salted meat aiming at reducing sodium content. To evaluate the effect of different salts on lipid oxidation thiobarbituric acid-reactive substances (TBARs) assay was performed along 180 days. Furthermore, ESI-MS/MS and GC analysis were conducted to detect and identify oxidized lipids, volatile compounds and free fatty acids profiles during the meat processing time. Lipid profiles from different salted meat demonstrated that CaCl_2_ salt have inducted more lipid oxidation when compared to the combination of NaCl and KCl salts, highlighting the implication of CaCl_2_ on increased lipolysis reactions. Moreover, the obtained results from both the analyses suggest that a combination of NaCl and KCl salts can be a good alternative for reducing the sodium content without compromising the quality of the salted meat.

## 1. Introduction

Salted meats are consumed and appreciated worldwide because of their unique sensory characteristics and shelf stable properties. The consumption is an excellent alternative to improve the nutritional status of people who lives in an area with deficiencies in the cold chain. However, despite all these benefits, these meat products have high sodium content and depend of good gastronomy practices to provide an adequate desalting step not always observed by consumers.

Salting is a traditional method of preservation of several meat products and undoubtedly appears as an important technology for the development of meat industry [1]. Many countries have traditional salted meat such as biltong in South Africa, jerked beef in Brazil [2], bresaola in Italy [3], and cecina in Spain [4]. Particularly, in Brazil, these products are consumed in a large scale being an important economical item for national meat industry also focused in exportation due to high acceptation sensory. In the context of public health, a significant part of population living in poorest regions finds their nutritional requirements of essential amino acids, minerals, mainly Fe and vitamins from B complex by jerked beef or charqui consumption [5]. The Brazilian meat industry has great interest in developing healthier salted meat products by sodium reduction giving consistent claims to support the consumer of this meat product in a healthy consumption in a current scenario.

Salting process is responsible for many changes in meat characteristics along drying and consequent reducing activity water. The most important are related to color, taste, proteolysis and lipolysis. NaCl is the most important ingredient used for the development of several functional and sensorial characteristics in meat products, through influence the water retention and binding capacity, modify the texture properties and provides salty taste [6], resulting in improved microbiological stability. 

A disadvantage of excess of sodium intake is increasing the risk of development of many health disorders such as cardiovascular diseases and hypertension [7,8,9]. However, to elaborate salted meat, higher amounts of NaCl are used during salting stages, which is unhealthy for consumption. Hence, an additional desalting step is required for the elaboration of salted meat. Taking into account the high sodium content, the partial NaCl replacement by others salts in the salting steps becomes a promising strategy for effective reduction of sodium in different meat products, but this reformulation can result in some technological challenges.

Meat lipids are mainly composed of triglycerides and phospholipids, which contain saturated fatty acids (SFAs), monounsaturated fatty acids (MUFAs) and polyunsaturated fatty acids (PUFAs). Triglycerides are storage lipids and are composed of three fatty acids esterified to glycerol and richer in SFAs, whereas phospholipids are often functional lipids prevalent in cell membranes and as such contain more PUFAs than triglycerides [10]. The content of unsaturated fatty acids will depend on the meat raw material, and the amount used in the processing [11] that could play a significant role on sensory properties of salted meats. Oxidation of unsaturated fatty acids can occur during processing and storage of meat and to a certain extent, it is a desirable phenomenon as it produces active compounds that influences flavor (e.g., aroma and taste) [12,13]. In the same way, along salted meat processing is also characterized by lipolysis that results from the breaking down of triglycerides and phospholipids through hydrolysis or by an enzymatic process, generates glycerol and FFA [14]. This phenomenon is intensely observed during the salting steps and storage, being affected by different salts [9]. FFA starts to accumulate as the process progresses and with an increased period of storage, later they tend to decrease due to the higher susceptibility to oxidation.

The reactions of lipolysis and lipid oxidation are interpreted as different phenomena and these reactions can be directly influenced by water activity (aw), pH, nitrite, metals, salts, and storage [14]. The salt added to elaborate meat products contribute and accelerate lipid oxidation, which is one of the main responsible for the quality losses along shelf life [15]. The catalytic role of NaCl may be due to the displacement of the iron ions from macromolecules altering the reactivity and distribution, thus increasing its catalytic activity. Furthermore, NaCl may form chelate complexes with ferric iron [16], which is suggested to be responsible for the catalysis of lipid oxidation in biological tissues. In the salting process, the temperature also can catalyze the lipid oxidation reaction [17]. An intense process of oxidation, however, could reach the level of rancidity, and the foodstuff would no longer be acceptable for human consumption. In addition, before such a condition is reached, lipid oxidation reactions could generate toxic compounds including cholesterol oxides and malonaldehyde, which are associated with increased risk of developing of certain types of cancer, heart disease and premature aging [18].

The role of NaCl in salted meats is important to maintain the typical identity of these products, and because of this, the reducing sodium by substitution by other salts is a great technological challenge and it has been intensively studied. However, the literature is very rich in studies on lipolysis of fermented meat products combined with dehydration but very poor or nonexistent regarding salted meat reactions. Thereby, the objective of the present study was to investigate the effect of NaCl reduction and substitution by KCl and CaCl_2_ blends on lipolysis, aiming at sodium reduction in salted meat.

## 2. Materials and Methods 

### 2.1. Treatments, Material and Additives 

The NaCl, KCl and CaCl_2_ salts used in salting step processes were food grade (Anidrol. Brazil). The bovine raw meat was the *biceps femoris* obtained from slaughterhouse with assured sanitary quality (Friboi, São Paulo, Brazil). The additives sodium erythorbate and sodium nitrite were donated by the company Kerry of Brazil. All the solvents as chloroform, methanol and xylene were HPLC grade purchased from Merck (Chile), and ultra-pure water was used in all experiments. Paraffin-embedded meat sample was dewaxed with hexane solvent followed by extraction of the lipids from the meat sample. Four treatments of salted meat were performed as described in Table 1. The NaCl replacement by KCl and CaCl_2_ was based on ionic strength of control treatment (100% NaCl), obtaining the same ionic strength in all treatments. 

### 2.2. Salted Meat Processing 

**Figure 1:**
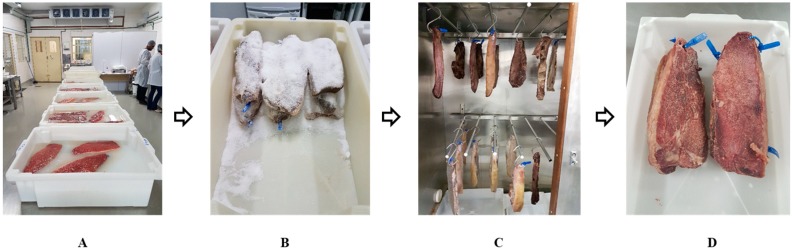
Processing steps for obtaining salted meat. **A**: wet salting; **B**: dry salting; **C**: maturation; **D**: final product.

The salted meat processing was performed according to previous published work [2]. In the wet salting, the raw bovine meat was immersed in a respective saturated solution with salts and additives (respective salt, 0.015% sodium nitrite and 0.05% sodium erythorbate) for 1 h as described in Table 1. After wet salting, the meat pieces were put in contact with respective salts for dry salting step (Table 1) for 144 h (6 days) in a cold chamber at 13 °C. After that, ripening was carried out in a controlled climatic chamber (Instala Frio, Curitiba, Brazil) with 55% humidity, 25 °C and 0.5 m/s forced air ventilation for 24 h (1 day). The final product was vacuum packed with polyethylene (Spel, São Paulo, Brazil) and stored at 25 °C. The processes were executed in the Pilot Plant of the Meat Area, Faculty of Food Engineering, University of Campinas. The process was carried out in triplicate with same technology and methodology in three different days, as depicted in Figure 1.

### 2.3. Lipid Oxidation 

The lipid oxidation of salted meat was measured by the amount of thiobarbituric acid-reactive substances (TBARs) as described in reference [19], using trichloroacetic acid instead of perchloric acid as the solvent. The results were expressed in g of malonaldehyde (MDA)/kg for each sample. The lipid oxidation was measured at 0, 45, 90, 135 and 180 days of storage in triplicate for each treatment in each process replicate.

### 2.4. Instrumental Color

Color measurements were determined with 20 mm aperture, D65 illuminant and 10° standard observer using Hunter Lab colorimeter (Colourquest II, Hunter Associates Laboratory Inc., Reston, VA, USA). L*, a* and b* color parameters were determined as an indicator of luminosity, red intensity, and yellow intensity, respectively. The whiteness index was calculated by the following equation:

100 − ((100 − L*) 2 + 2a* + 2b*)^1/2^ (1)

The color parameters were measured at 0, 45, 90, 135 and 180 days of storage in triplicate for each treatment in each process replicate.

### 2.5. Lipolysis Analysis

#### 2.5.1. Formalin-Fixed Paraffin-Embedded (FFPE) Sample Preparation

The samples were fixed according to Bancroft [20] with some modifications. The salted meat samples were cut and placed in a flask with buffered formaldehyde solution (4%, phosphate buffer 0.075 M, pH 7.3) 1:20 ratio of salted meat formaldehyde solution, remaining in the solution for 48 h for tissue fixation. After that, the tissues were placed in a cassette with 11% formic acid solution overnight, then, the samples were put in 70% ethanol solution. The following steps were: 1 h in a solution of 95% ethanol and 5% methanol, 4 times of 90 min with absolute ethanol, 2 times of 1 h with xylene, and finally 2 times of wax (Paraplast Plus, McCormick) at 58 °C for 1 h.

#### 2.5.2. Lipids Extraction

Primarily, salted meat samples were dewaxed as reported by Wojakowska [21]. In brief, FFPE salted meat samples were gently immersed in 100% xylene for 10 min, which was kept at 60 °C for efficient paraffin solvation. After that, the samples were rehydrated and were subsequently used for lipids extraction. Folch protocol [22] was used for extracting total lipids from salted with modifications. Salted meat samples were sonicated with 10 mL of chloroform/methanol mixture (2:1) for 30 min. After homogenization, equal volumes of chloroform and water were added to the extract so that there was phase separation. The lower phase was collected into a test tube and the upper phase was taken again for washing with 2 mL of the solvent mixture; after separation of the extract lower phase was combined with the first extract. This process was repeated for 3 times and all the organic extracts were pooled together and the solvent was removed by evaporating under vacuum in a rotary evaporator and dried lipids were stored at −20 °C until use. 

#### 2.5.3. Electrospray Ionization Mass Spectrometer (ESI-MS) Analysis

Lipid analysis was performed on an electrospray ionization-tandem mass spectrometer (ESI-MS/MS) with a linear ion-trap mass analyzer (Amazon, Bruker) equipped with a Hamilton syringe pump and an electrospray source. Samples were prepared by dissolving 1 mL of the lipid sample in 100 mL of methanol. The sample was injected at an infusion rate of 5 µL/min, the ion spray voltage was set at −4.5 kV and the source temperature were at 220 °C for both positive and negative ionization modes. MS/MS experiments were conducted manually for identification of lipid species with nitrogen as collision gas and collision energy of ~50 eV. Data analysis software package (Bruker Daltonics) was used to collect full scan spectra over the range of *m/z* 100–1200 and the obtained raw data was preprocessed for smoothening baseline subtraction peak picking, and deconvolution if needed.

#### 2.5.4. Volatile Compound Profile 

The extraction of the volatile compounds was performed using solid-phase microextraction (SPME), followed the conditions described by Domínguez [23]. For headspace SPME (HS-SPME) extraction, 1 g of each sample was weighed in a 20 mL vial, after being ground using a commercial grinder. The conditioning, extraction and injection of the samples were carried out with an autosampler PAL-RTC 120. The extractions were performed at 37 °C for 30 min, after equilibration of the samples for 15 min at the temperature used for extraction, ensuring a homogeneous temperature for sample and headspace. Once sampling was finished, the fiber was transferred to the injection port of the gas chromatograph–mass spectrometer (GC–MS) system. A gas chromatograph 7890B (Agilent Technologies, Santa Clara, CA, USA) equipped with a mass selective detector 5977B MSD (Agilent Technologies) and a DB-624 capillary column (30 m, 0.25 mm i.d., 1.4 μm film thickness; J&W Scientific, Folsom, CA, USA) was used for volatile analysis. Compounds were identified by comparing their mass spectra with those contained in the NIST14 (National Institute of Standards and Technology, Gaithersburg) library, and/or by comparing their mass spectra and retention time with authentic standards (Supelco, Bellefonte, PA, USA), and/or by calculation of retention index relative to a series of standard alkanes (C5–C14) (for calculating Linear Retention Index, Supelco 44585-U, Bellefonte, PA, USA). The results are expressed as area units (AU) of the Quantifier Ion × 10^4^/g of sample.

#### 2.5.5. Free Fatty Acids Profile 

Total lipids were extracted from 5 g of salted meat sample, the methodology was performed according to Folch [22]. Free fatty acids (FFA) were separated using NH_2_-aminopropyl mini-columns as described by Regueiro [24]. 50 mg of the extracted lipids were transesterified with a solution of boron trifluoride (14%) in methanol, and the FAMEs were stored at −80 °C until chromatographic analysis. Separation and quantification of FAMEs was carried out using a gas chromatograph GC-Agilent 6890N (Agilent Technologies, Madrid, Spain) equipped with a flame ionization detector and an automatic sample injector HP 7683, and using a Supelco SPTM-2560 fused silica capillary column (100 m, 0.25 mm i.d., 0.2 μm film thickness, Supelco Inc., Bellafonte, PA, USA). Chromatographic conditions were as follows: initial oven temperature of 120 °C (held for 5 min), first ramp at 2 °C/min to 170 °C (held for 15 min), second ramp at 5 °C/min to 200 °C (held for 5 min) and third ramp at 2 °C/min to final temperature of 235 °C (held for 10 min). The injector and detector were maintained at 260 and 280 °C, respectively. Helium was used as carrier gas at a constant flow-rate of 1.1 mL/min, with the column head pressure set at 35.56 psi. One μL of solution was injected in split mode (1:50). The fatty acids were quantified using nonadecanoic acid methyl ester, at 0.3 mg/mL, as internal standard that was added to samples prior to fat extraction and methylation. Identification of fatty acids was performed by comparison of the retention times with those of known FAME standard and the results expressed as g/100 g of total fatty acids.

#### 2.5.6. Statistical Analysis 

In each process, at least three samples were collected for each analysis. The results expressed in this work are averages obtained from all data. The commercial software Statistica v.8 (Statsoft Inc., Tulsa, OK, USA) was used to perform general linear models analysis and Tukey’s test (*p < 0.05*) considering the treatments as a fixed effect and the replicates as a random effect using 5% of significance. 

## 3. Results

### 3.1. Lipid Oxidation (TBARs)

Lipid oxidation is the major reason for deterioration of meat and meat products promoting rancidity, loss of essential fatty acids, undesirable odor and texture, besides production of toxic compounds [25,26,27]. Salted meats products are particularly susceptible to the rapid development of lipid oxidation due to high NaCl concentration, which is considered as a potent pro-oxidant and has a low or intermediate water activity [28]. The TBARs values obtained along the shelf life of reformulated salted meat with KCl and CaCl_2_ blends are shown in Table 2. 

### 3.2. Lipolysis 

As discussed earlier, the phenomena of lipid oxidation or lipid peroxidation are mostly observed in polyunsaturated fatty acids such as omega-3 fatty acids (α-linolenic acid, eicosapentaenoic acid and docosahexaenoic acid) and omega-6 fatty acids (linoleic acid, arachidonic acid and docosapentaenoic acid). These PUFAs produces a wide variety of oxidation products, primary products of lipid peroxidation are lipid hydroperoxides that on further oxidation forms different aldehydes such as malonaldehyde (MDA), propanal, hexanal, 4-hydroxynonenal (4-HNE) and other F2-isoprostanes such as 8-iso-prostaglandin F2α (8-iso-PGF2α).

**Figure 2:**
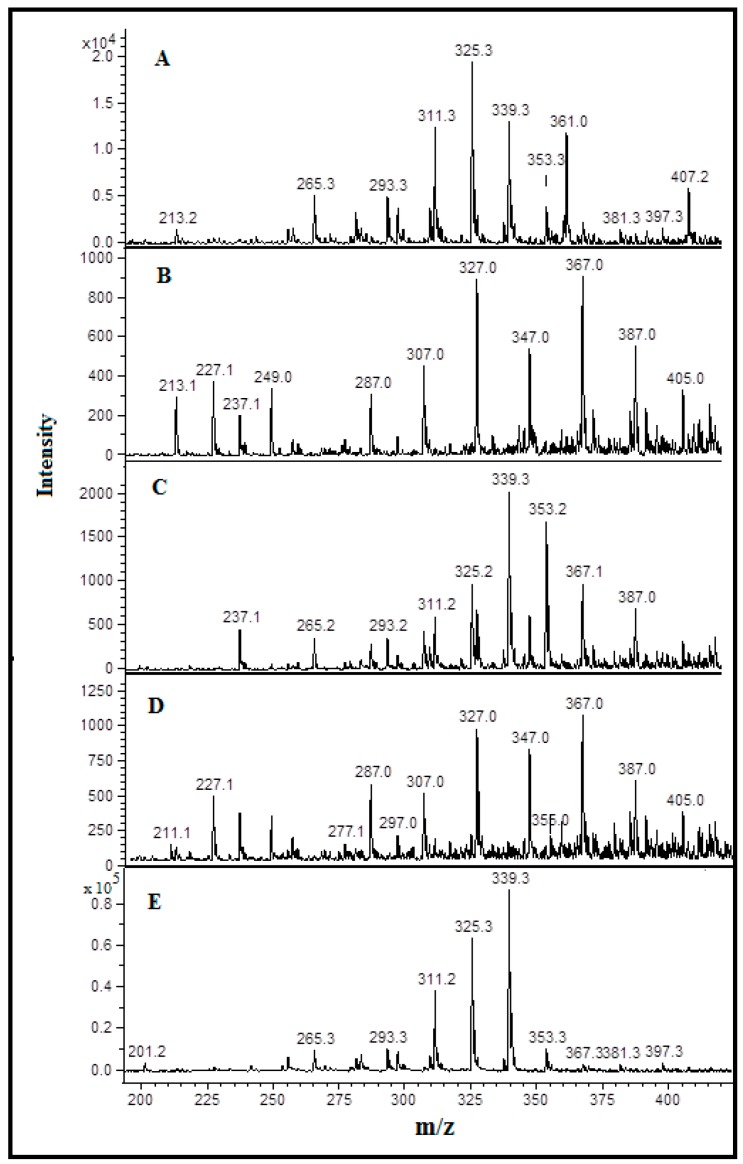
Lipid profiles of salted meats at initial days of storage i.e., at T0, (**A**) control; (**B**) FC1: 100% NaCl; (**C**) F1: 50% NaCl + 50% KCl; (**D**) F2: 50% NaCl + 50% CaCl_2_; (**E**) F3: 50% NaCl + 25% KCl + 25% CaCl_2_ obtained in ESI-MS in negative ion mode.

Thus, trying to explain the effect of different salts on salted meat during lipolysis and lipid oxidation, it was employed ESI-MS without any derivatization step to analyze lipid composition and detecting and identifying the oxidized lipids. Primarily, lipid profiles of different salted meat treatments were obtained by ESI-MS in positive and negative modes at different days of storage. The full scan spectra in negative ion mode (Figure 2) demonstrates that the total fatty acid composition of FC1, F1, F2 and F3 samples at initial days of storage (T0) has majorly PUFAs especially, linolenic acid (18:2n-6), arachidonic acid (AA) (20:4n-6), eicosapentaenoic acid (20:5n-3), and docosahexaenoic acid (22:6n-3). 

### 3.3. Detection and Identification of Oxidized Phospholipids 

Detection and identification of oxidized phospholipids and fatty acids were performed by selecting particular oxidized phospholipid of interest and then carrying out fragmentation to obtain structural information by lipidomics protocol. 

The oxidation of AA was identified by selecting the precursor ion of *m/z* 353, which was readily detected with good intensity in the full scan spectra. The characteristic fragment ions for the identification of these oxidized fatty acids and their possible structures are summarized in Table 3.

**Figure 3:**
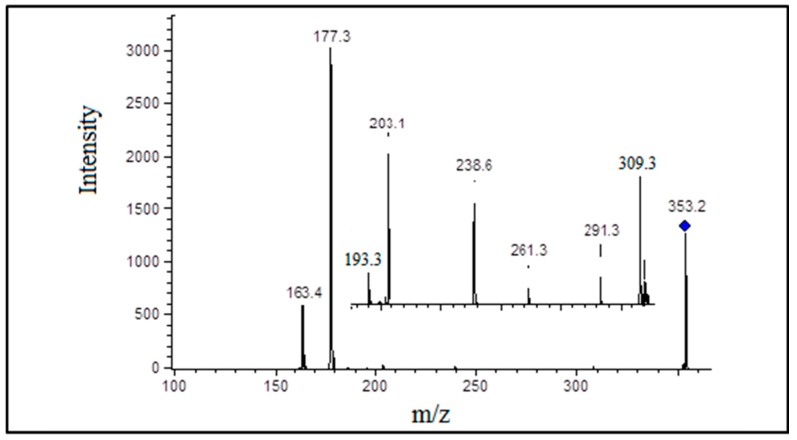
Structural identification of oxidized arachidonic acid by ESI-MS/MS fragmentation pattern.

Selective fragmentation of the precursor ion of *m/z* 353 (Figure 3) exhibited fragment ions of *m/z* 237 and 309, which are the signature peak of PGF2α, and an ion with *m/z* value of 193 corresponding to decarboxylation of the peak of *m/z* 237. Presence of the product ion at *m/z* 193 in the MS/MS spectra of *m/z* 353 confirms oxidation of arachidonic acid (20:4). 

### 3.4. Volatile Compound Profile 

**Figure 4:**
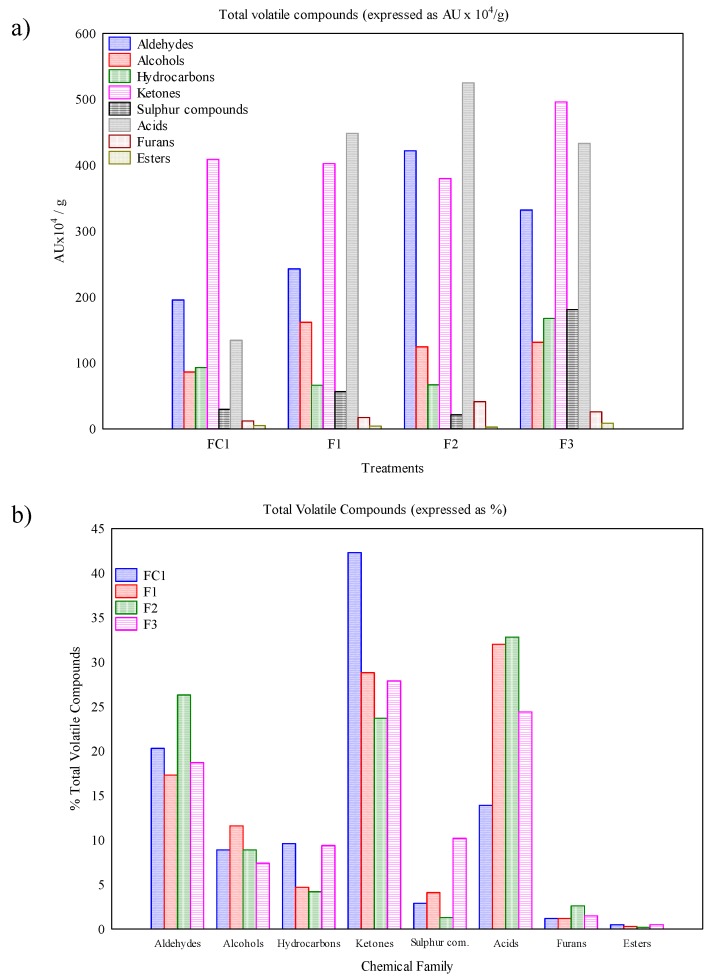
Graphic representation of the volatile compounds expressed as AU × 10^4^/g. (**a**) % of total volatile compounds, (**b**) from each chemical family detected on salted meat. FC1: 100% NaCl; F1: 50% NaCl + 50% KCl; F2: 50% NaCl + 50% CaCl_2_; F3: 50% NaCl + 25% KCl + 25% CaCl_2._

The volatile profile of salted meat is shown in the Table 4 (expressed as UA × 10^4^/g of dry matter). A total of 57 volatile organic compounds (VOC) of 8 different chemical groups were separated and identified in the meat samples. These groups were hydrocarbons (17 compounds), alcohols (11), aldehydes (8), ketones (8), acids (6), sulphur compounds (4), esters (2) and 1 furan. The major volatile compounds identified in the present study are in line with those reported in the literature for other dry-cured products [23,29,30]. Figure 4 shows the total VOC from each chemical family (both, as AU × 10^4^/g and as percentage of total volatile compounds) in the 4 batches of salted meat analyzed. Statistical analysis using VentVed showed significant differences in the VOC contents between batches. The F2 and F3 batches exhibited higher values of total VOC (1603 and 1777 AU × 10^4^/g, respectively; data not shown) than F1 (1401 AU × 10^4^/g; data not shown) that present intermediate values and FC1 (967 AU × 10^4^/g; data not shown), which had the lowest value. 

### 3.5. Free Fatty Acids Profile 

Lipolysis generates FFA, and after the lipid oxidation process, produces several volatile compounds such as methyl ketones, alcohols and aldehydes [13]. According to Lorenzo and Carballo [31], each individual free fatty acid should be the balance result between its release from phospholipids, triglycerides and its oxidative degradation. The profile of FFA can be verified in Table 5. 

The main FFAs in all salted meat treatments were oleic acid (C18:1n-9), palmitic acid (C16:0) and stearic acid (C18:0). The FFA profile of these salted meat treatments is similar as described by other authors in others dry-cured meat products [31,32,33].

## 4. Discussion

According to Table 2, the highest values of TBARs were found in 45 days of storage in all treatments and then generally decreased, however, after 180 days of storage, a further increase of malonaldehyde occurred in all treatments. These results can be explained by the precise identification of the malonaldehyde in the initial and propagation stages [34].

The replacement of 50% NaCl by KCl (F1: 50% NaCl + 50% KCl) resulted in the lowest malonaldehyde content (*p* < 0.05) after 90 days of storage compared to other treatments. By other hand, the salted meats containing CaCl_2_ (F2: 50% NaCl + 50% CaCl_2_ and F3: 50% NaCl + 25% KCl + 25% CaCl_2_) presented the highest values of malonaldehyde (*p* < 0.05) in the first 90 days of storage if compared to FC1 (100% NaCl) and F1 (50% NaCl + 50% KCl) treatments. Dos Santos [35] obtained similar results of increase of malonaldehyde values in salami with replacement of 50% NaCl by 50% CaCl_2_ and also reported that the addition of CaCl_2_ increase the lipid oxidation by generation of hexanal and (E)-hept-2-enal and other volatiles during processing and storage of fermented sausages [36]. These results of TBARs demonstrate a greater lipid oxidation capacity of CaCl_2_ when compared to NaCl and KCl during 180 days of storage, in agreement to Vidal [2] who founded similar results of malonaldehyde values during elaboration of jerked beef added of NaCl, KCl and CaCl_2_.

**Scheme 1:**
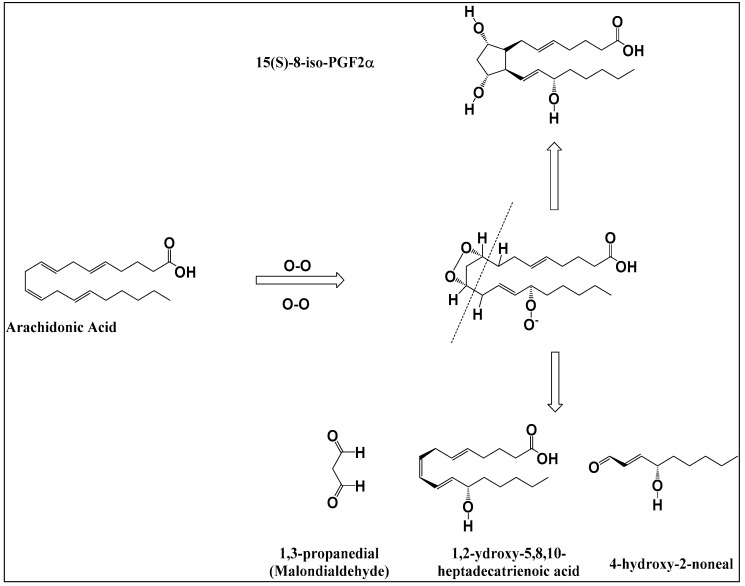
Proposed mechanism for the formation of 1,3-propanedial (malondialdehyde) by 1,2-hydroxy-5,8,10-heptadectrienoic acid, 4-hydroxy-2-noneal and 15(s)-8-iso-PGF2α from peroxidation of Arachidonic acid (AA).

To understand the mechanism of formation of primary and secondary products of lipid peroxidation from fatty acids, an example of arachidonic acid undergoing lipid peroxidation is shown in Scheme 1.

**Figure 5:**
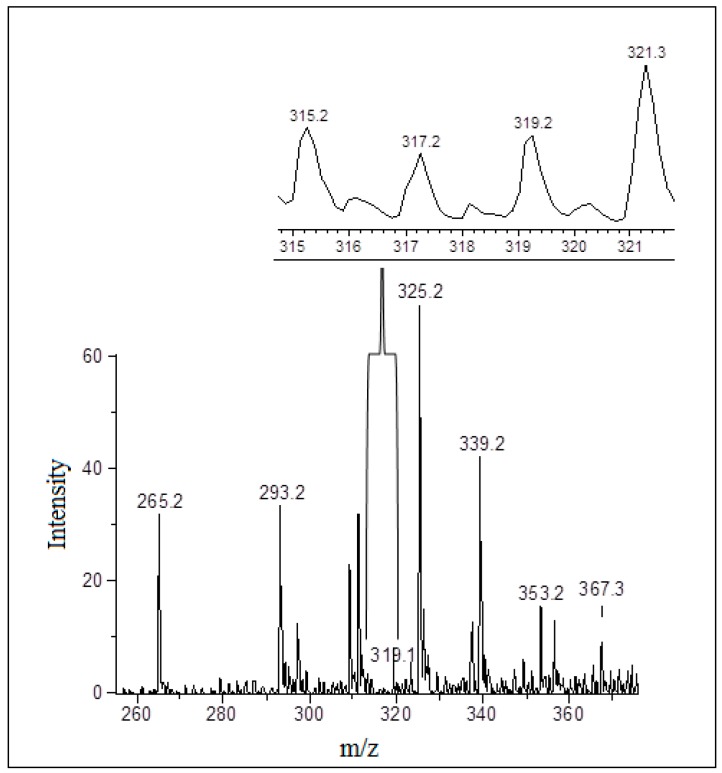
Detection of oxidized polyunsaturated fatty acid from ESI-MS spectra of FC1 T180 sample at *m/z* range of 250–400 in negative ion mode. Precursor ions of *m/z* 293, 295, 317 and 319 are characteristic peaks for oxidation of linoleic and arachidonic acids.

**Figure 6:**
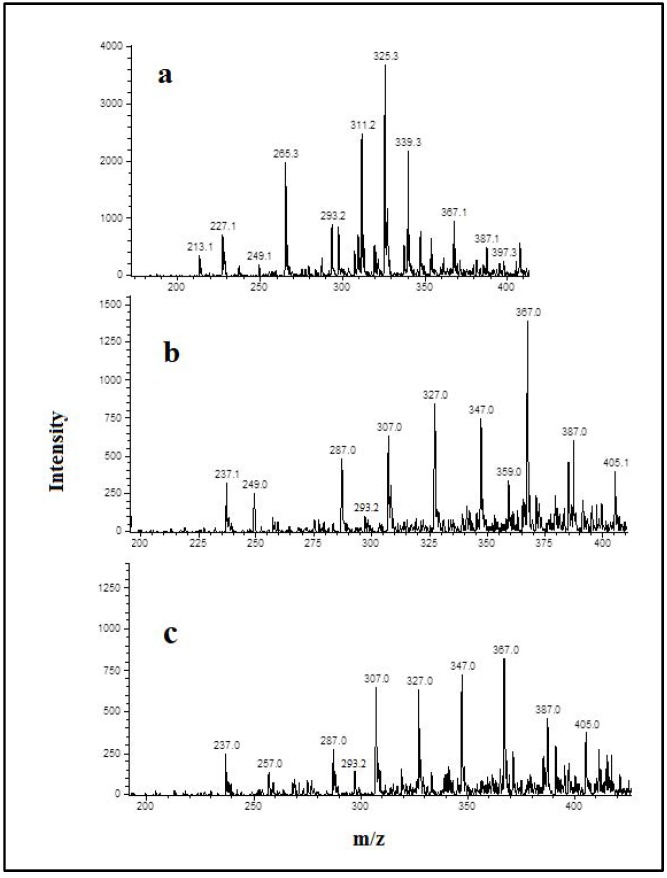
Detection of oxidized polyunsaturated fatty acid from ESI-MS spectra of samples (**a**) F1 T180, (**b**) F2 T180 and (**c**) F3 T180 at *m/z* range of 250–400 in negative ion mode. In the spectra, it demonstrates characteristic peaks for oxidation of linoleic acid with peak ions *m/z* 293 and 295, respectively.

Thus, it can be assumed that arachidonic acid (AA, 20:4n-6) with *m/z* 303 present at the sn-2 position in phospholipid could have undergone lipolysis and released AA as a free fatty acid, which on further lipid peroxidation could have formed PGF2α product, as depicted in Figure 3. A detailed mechanism demonstrating the formation of secondary products of lipid peroxidation from AA is shown in Scheme 1. Similarly, various oxidized peaks were detected in the full scan spectra in negative ion mode from different salted meat samples (Figure 5 and Figure 6), for instance, peak *m/z* 293 is observed due to oxidation of α-linolenic acid (18:3n-3), which may form 9-oxo or 13-oxo octadecadienoic acid. Likewise, peak of *m/z* 295 was observed due to oxidation of linoleic acid (18:2n-6) and peaks of *m/z* 317 and 319 were found due to oxidation of arachidonic acid (20:4n-6), respectively. 

The products of lipid peroxidation were extensively studied by Esterbauer and his colleagues in the 80s. In their work, they found that MDA appears to be the most mutagenic product of lipid peroxidation, whereas 4-HNE is the most toxic compound [37,38,39,40]. Further advanced studies on the identification of positional isomers of hydroxy, hydroperoxy and keto phospholipids including those derived from the oxidation of PUFAs were reviewed [41]. From the reported studies, it was evident that secondary products of lipid peroxidation appear to be carcinogenic due to excessive lipid oxidation. Hence, the reduction of the NaCl, main source of sodium, during meat processing is quintessential.

Some MUFAs such as oleic acid (18:1) and SFAs like myristic acid (14:0), as well as arachidic acid (20:0) were observed in samples with 0 day of storage. This could be due to the increased enzymatic activity of phospholipase enzymes, which could have released FFA from Sn-2 position of phospholipids [42,43]. However, with an increased period of storage i.e., at 180 days, it was observed that FC1 T180 and F2 T180 samples have demonstrated changes in the free fatty acids composition.

It was also observed that many peaks corresponding to PUFAs decreased. Perhaps, it could be due to the effect of CaCl_2_ salt on lipolysis that produced oxidation of FFA. However, the effect of salt directly or indirectly plays an important role in the generation of volatile and non-volatile compounds that enhance the flavor of the meat [44,45,46], which are in agreement with previous reported studies. It was observed that *semimembranosus* and *biceps femoris* muscle from ham with higher salt content showed a marked decrease in the total fatty acid content especially PUFAs [47]. Similar effects were observed in ham during lipolysis [42,43]. In order to confirm if really lipid peroxidation was occurring during a prolonged period of storage, we studied the full scan spectra of all treated meat samples obtained in negative ion mode by ESI-MS.

Regarding volatile compounds, NaCl replacement increased the total VOC. In similar way, Armenteros [48] found in ham samples that the replacement of NaCl by a blend of chloride salts (potassium, calcium and magnesium) resulted in an increase of total VOC. In contrast, Domínguez [49] described the highest values in control samples, mainly due to the highest amounts of hexanal found in this batch. Not only the total VOC, but also the volatile profile was also affected by the salt treatment. In FC1 and F3 batches, the major VOC were the ketones (Figure 4), representing 42.3% and 27.9% of total VOC, respectively, while in F1 and F2 batches, acids were the major group (about 32% in both treatments). In contrast with our values, other authors found that aldehydes were the more abundant volatiles in meat products [23,48,50,51]. However, not all researches show the same results. In fact, ketones were also reported as the major chemical group in sausages [23] and also in cured loin [52], while acids were found the main group in cured loin [23]. As could be seen in the Figure 4, although there are differences among treatments, the major volatile groups in all samples were ketones, acids and aldehydes (in different proportions). The major individual VOC, except for FC1 treatment, was butanoic acid (352–447 AU × 10^4^/g) followed by hexanal (183–299 AU × 10^4^/g) and acetoin (171–204 AU × 10^4^/g). Contrary, in the FC1 samples, the major VOC was acetoin (145 AU × 10^4^/g), followed by hexanal (135 AU × 10^4^/g) and butanoic acid (101 AU × 10^4^/g). High amounts of these 3 compounds were found in other dry-cured products [23]. Therefore, it seems that these compounds had an important influence in the aromatic characteristics of the salted meat. 

However, the major differences among control batch (FC1) and the other treatments were observed in the content of acids, ketones and aldehydes, although the other chemical families also showed minor differences. With this regards, it is well known that the compounds derived from lipid oxidation reactions are aldehydes, ketones, some carboxylic acids and also alcohols [53]. Therefore, as will be discussed below, it seems that the compounds derived from lipid oxidation had high influence in total volatile compounds content in salted meat. 

A total of 6 organic acids were identified in samples. In this case, all experimental treatments had higher values than samples from control batch. The major amount of total acids was found in F2 (525 AU × 10^4^/g) followed by F1 (449 AU × 10^4^/g), F3 (433 AU × 10^4^/g) and finally FC1 (135 AU × 10^4^/g). The major acid, as commented above, was butanoic acid, followed by acetic and hexanoic acids. In the present study, the content of these three acids was higher in the samples with partial replacement of NaCl than in control. These findings were also reported in dry-cured loin [49]. Additionally, all these acids were also found in dry-cured loin [32,52] and hams [54]. In contrast with our results, Armenteros [48] reported that the replacement of NaCl by other chloride salts resulted in a decrease of total acids content. The main origin of the acetic acid is frequently related with carbohydrate fermentation and Maillard reaction [23,54]. In salted meat as performed in this study, it makes sense, considering the last processing step when the dry meat is put under 35–40 °C to improve the ripening. In this condition, the Maillard reactions can be favored. Other straight-chain carboxylic acids are derived from the hydrolysis of lipids (triglycerides and phospholipids) [55]. This fact could explain the differences found between studies and meat products, due mainly to the different ingredients and dry-cured conditions. Additionally, the content of NaCl and the partial replacement by other salts also affect the activity of the enzymes and therefore, the release of precursor compounds for the formation of VOC.

Regarding ketones content, acetoin showed the major values, following by 2,3-butanedione. Also, important amounts of 2-butanone and 2-heptanone were found. Acetoin also was the major ketone in other meat products as loin, salchichón, shoulder and chorizo [23]. The highest contents of acetoin were observed in samples from F1 and F3 samples, while the lowest values were found in control batch. The content of 2-butanone and 2-heptanone, both related with lipid oxidation, was higher in F2 and F3 treatments (salt blends that include calcium in their formulation) than in F1 and FC1. The origin of ketones can be diverse. Linear ketones, especially 2-ketones and methyl ketones arise from the oxidation of free fatty acids [53,56], while other ketones such acetoin are formed through Maillard reactions [54].

Aldehydes represented around 20% of total VOC in all batches. However, if we observe the Figure 4, it is clear that their content (AU × 10^4^ /g) suffered a significant increase in F2 and F3 batches, in comparison with F1 and control samples. In fact, the major increment was detected in F2 samples, which contain the highest amount of calcium in their formulation. In all samples, the major aldehyde was hexanal, followed by pentanal and heptanal. In dry-cured lacón also was reported that these 3 compounds were the major aldehydes detected in the final product [49]. Additionally, there are other researches that found hexanal was the major aldehyde in dry-cured products [48,57,58]. The content of other aldehydes also increased with the partial replacement of NaCl. The amount of octanal, pentadecanal and propanal, 2-methyl were higher in F2 and F3 samples than in control samples. It is well known that hexanal, and pentanal deriving from the oxidation of linoleic, linolenic and arachidonic fatty acids, while heptanal, octanal and nonanal come from oleic acid autoxidation [58,59]. Additionally, aldehydes due to their low odor threshold values, have and important role in the aroma of dry-cured meat products [51,53]. However, the presence of some aldehydes is not always related with quality decrease. The aldehydes derived from oleic acid oxidation, such as heptanal, octanal and nonanal have been related to pleasant meaty notes [23].

Finally, other important VOCs that had influence in the final aroma were also found in the present study. These compounds were all related with lipid oxidation. To this regard, the content of linear hydrocarbons as pentane, heptane, octane, dodecane and tridecane was higher in samples from treatments F2 and/or F3 than in F1 and control samples. In the same way, the content of lipid oxidation-derived alcohols such as 1-propanol, 1-butanol, 1-octen-3-ol showed the highest values in F2 samples (which contains the major amount of calcium in their formulation), and the content of furan, 2-penthyl, also derived from the oxidation of linoleic acid was higher in the samples from F2 and F3 than in the other 2 batches. 

As a general comment, the F2 and F3 batches showed the highest contents of lipid-derived VOC, while F1 had intermediate values, in comparison with control samples. It is well known that during processing chemical reactions and enzyme activity are involved in the generation of volatile compounds affecting the sensory properties of dry-cured meat products. There are multiple researches that studied the influence of salt replacement on lipolytic and oxidative processes, which are directly related with volatile generation. The high content of NaCl influences the progression lipolytic/oxidation reactions during the ripening and dry-curing stages [15,60]. Therefore, it is expected that its partial replacement exerted a significant effect on the final content of volatile compounds. 

Furthermore, the replacement of NaCl also influenced the free fatty acids contents. The use of blends of substitutes salts (KCl and CaCl_2_) affected the proportion of: caprylic acid (C8:0), palmitic acid (C16:0), heptadecanoic acid (C17:0), stearic acid (C18:0), oleic acid (9t-C18:1), cis-vaccenic acid (C18:1n-7), linoleic acid (C18:2n-6), γ-linolenic (C18:3n-6), α-linolenic (C18:3n-3), 9-cis 11-trans-octadecadienoic (9c,11t-C18:2 (CLA)) and eicosapentaenoic acid (C20:5n-3).

Moreover, different authors found an increase in the fatty acid release and FFA content as results of NaCl replacement, mainly in samples salted with blends that include chloride salts with divalent ions [61]. In similar way to lipolysis, other authors found that lipid oxidation was also higher in samples salted with blends containing divalent ions than samples salted with NaCl [2,62], which indicate that these salts favored the progression of oxidative reactions in the lipids of dry-cured meats. Therefore, the reduction of NaCl proportion in the salting step increased lipolytic and oxidative processes. The role of divalent salts in both lipolysis and lipid oxidation remains unclear due to contrasting results observed among studies [30,61]. However, it seems that the use of CaCl_2_ in the blend composition increase lipid oxidation. Furthermore, another study concluded that the amount of CaCl_2_ used was important because larger amount favored the lipid oxidation [63], which is in agreement with our results. The samples containing CaCl_2_ in the salt blend (F2 and F3) showed the highest TBARs values (1.10 and 1.74 mg MDA/kg, respectively), in comparison with the values of the other batches (about 0.9 mg MDA/kg). Therefore, it easy to conclude that the main differences found in volatile contents and profiles among the samples analyzed in the present study are due to the differences in the oxidation processes between the different batches.

## 5. Conclusions

Altogether, our results showed that the partial replacement of NaCl by KCl and CaCl_2_ influenced the lipolysis reactions and lipid profile in reduced sodium salted meats. CaCl_2_ promoted the most several changes in lipid oxidation along shelf life (180 days) being responsible for the highest values of malonaldehyde. The total volatile compounds, volatile profile and free fatty acids profile were affected by salt used in salting steps. Ketones, acids and aldehydes were reported as the major groups of volatile compounds founded in different proportions and oleic acid (C18:1n-9), palmitic acid (C16:0) and stearic acid (C18:0) were the main free fatty acids founded in all salted meat treatments. Considering the differences of TBARs values among treatments and all the results present in this study, it is evident the highest oxidative capacity and impact of CaCl_2_ on lipid profile compared to NaCl and KCl in salted meat treatments at same ionic strength.

## Figures and Tables

**Table 1:** Salts and additives used to perform the salted meat treatments.

Treatments	NaCl (%)	NaCl (mg) *	KCl (%)	KCl (mg) *	CaCl_2_ (%)	CaCl_2_ (mg) *
FC1	100	1000	-		-	
F1	50	441	50	560	-	
F2	50	614	-	-	50	387
F3	50	513	25	326	25	162

The amount of salt added was based on the ionic strength, all treatments obtained the same ionic strength. * Salt proportion added according to ionic strength, for each 1000 mg of bovine raw meat was utilized 2000 mg of salt.

**Table 2:** Malonaldehyde (mg/kg) values in salted meat treatments during storage.

Treatments	0 Day	45 Days	90 Days	135 Days	180 Days
FC1	0.95 ^cC^	1.61 ^cA^	0.80 ^bC^	0.75 ^aC^	1.32 ^aB^
F1	0.88 ^cB^	1.78 ^cA^	0.35 ^cC^	0.50 ^bC^	0.71 ^cB^
F2	1.10 ^bB^	3.58 ^aA^	1.09 ^aB^	0.68 ^abC^	1.26 ^aB^
F3	1.74 ^aB^	2.40 ^bA^	1.20 ^aC^	0.38 ^cD^	0.95 ^bC^
Standard error	0.06	0.15	0.06	0.03	0.05

Values are means. ^a, b, c, d^ Values in the same column with the same lowercase letters do not differ significantly (*p* < 0.05) according to Tukey’s test. ^A,B,C,D^ Values in the same line with the same capital letters do not differ significantly (*p* < 0.05) according to Tukey’s test. FC1: 100% NaCl; F1: 50% NaCl + 50% KCl; F2: 50% NaCl + 50% CaCl_2_; F3: 50% NaCl + 25% KCl + 25% CaCl_2_.

**Table 3:** Structures of the oxidized polyunsaturated fatty acids with their precursor and their characteristic fragment ion masses.

Carbon Annotation	Compound	Precursor (*m/z*)	Product (*m/z*)	Chemical Structure
18:2	LA	279	261	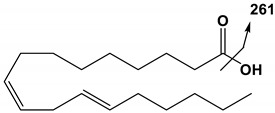
18:2	9-HODE	295	171	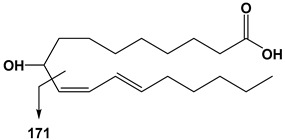
18:2	13-HODE	295	195	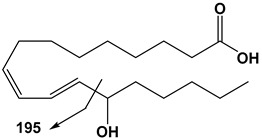
18:3	9-OxoODE	293	185	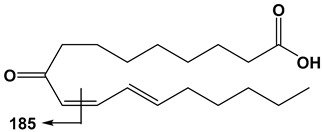
18:3	13-OxoODE	293	113	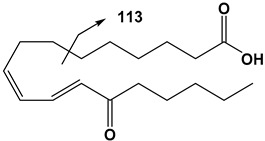
20:4	AA	303	259	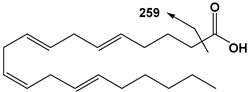
20:4	15-HETE	319	175	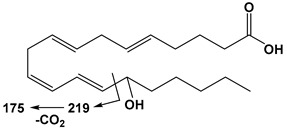
20:4	PGF2α	353	193	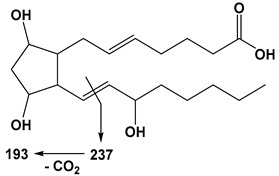
20:4	5-OxoETE	317	203	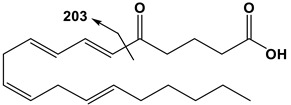
20:4	12-OxoETE	317	153	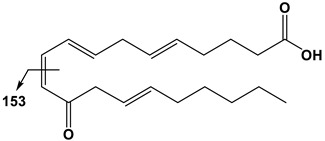
20:4	15-OxoETE	317	113	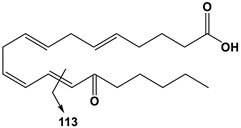

Lists the compounds that were detected in the ESI-MS spectra of lipids. the first column represents the carbon annotation of fatty acids. Second column shows the different fatty acids that are oxidized. Third column provides their masses and the fourth column shows their fragmented ions and the fifth column represent the structure of the oxidized fatty acids and the formation of the product ions by fragmentation. LA, linolenic acid; 9-HODE, 9-hydroxy linolenic acid; 13-HODE, 13-hydroxylinolenic acid; 9-OxoODE, 9-oxo-10E,12Z-octadecadienoic acid; 13-OxoODE, 13-Oxo-9,11-octadecadienoic acid; AA, arachidonic acid; 15-HETE, 15-hydroxy-5Z,8Z,11Z,13E-eicosatetraenoic acid; PGF_2α_ prostaglandin F2 alpha; 5-OxoETE, 5-oxo-6E,8Z,11Z,14Z-eicosatetraenoic acid; 12-OxoETE, 12-Oxo-5Z,8Z,10E,14Z-eicosatetraenoic acid; 15-OxoETE, 15-Oxo-5Z,8Z,11Z,13E-eicosatetraenoic acid.

**Table 4:** Volatile compounds (expressed as area units (AU) of quantifier ion × 10^4^/g) of salted meat (0 day of storage).

Volatile Compound	LRI	*m*/*z*	Treatments			
			FC1	F1	F2	F3
Glycidol	506	44	8.39 ± 0.52 ^b^	5.04 ± 1.02 ^b^	14.08 ± 1.54 ^a^	14.89 ± 1.70 ^a^
Methanethiol	509	48	0.41 ± 0.06 ^b^	0.93 ± 0.06 ^b^	8.39 ± 1.42 ^a^	6.80 ± 1.23 ^a^
Pentane	522	42	8.28 ± 0.26 ^a^	2.39 ± 1.05 ^b^	4.06 ± 2.53 ^ab^	7.93 ± 1.78 ^a^
Acetone	533	58	137.24 ± 38.23	112.18 ± 14.35	113.24 ± 30.45	164.53 ± 37.41
Dimethyl sulfide	535	62	3.74 ± 0.10 ^a^	3.50 ± 0.11 ^a^	1.34 ± 0.13 ^b^	3.13 ± 0.21 ^a^
Carbon disulfide	538	76	24.85 ± 0.31 ^b^	39.13 ± 3.73 ^a^	14.80 ± 3.96 ^c^	44.22 ± 2.22 ^a^
Propanal, 2-methyl-	562	72	1.30 ± 0.07 ^c^	1.36 ± 0.02 ^c^	4.82 ± 0.78 ^b^	5.85 ± 0.30 ^a^
1-Propanol	577	59	1.30 ± 0.24 ^bc^	1.74 ± 0.04 ^b^	3.16 ± 0.25 ^a^	0.92 ± 0.31 ^c^
2,3-Butanedione	593	86	108.44 ± 3.27 ^a^	70.70 ± 2.12 ^c^	54.22 ± 1.25 ^d^	81.52 ± 1.24 ^b^
2-Butanone	599	72	10.34 ± 1.10 ^b^	9.64 ± 3.16 ^b^	32.66 ± 6.58 ^a^	35.05 ± 8.01 ^a^
Hexane, 2,2-dimethyl-	665	57	34.93 ± 0.77	34.11 ± 1.14	27.10 ± 9.37	31.50 ± 1.68
Formic acid, 2-propenyl ester	682	57	1.90 ± 0.59 ^b^	1.97 ± 0.21 ^b^	1.87 ± 0.49 ^b^	4.61 ± 1.00 ^a^
Heptane	685	71	0.72 ± 0.10 ^b^	1.06 ± 0.01 ^b^	1.85 ± 0.06 ^a^	1.90 ± 0.19 ^a^
Acetic acid	704	60	10.31 ± 0.37 ^c^	31.86 ± 9.63 ^b^	39.78 ± 6.43 ^ab^	46.79 ± 35.52 ^a^
1-Butanol	718	56	1.86 ± 0.27 ^b^	2.57 ± 0.51 ^ab^	4.41 ± 0.97 ^a^	3.11 ± 0.75 ^ab^
Pentanal	739	58	15.71 ± 0.63 ^c^	11.24 ± 0.70 ^c^	29.86 ± 3.25 ^b^	44.44 ± 4.05 ^a^
Disulfide, dimethyl	793	94	1.09 ± 0.32 ^b^	13.22 ± 6.32 ^b^	4.98 ± 2.27 ^b^	95.68 ± 6.9 ^a^
Acetoin	799	88	145.28 ± 4.40 ^b^	204.05 ± 22.72 ^a^	171.95 ± 1.26 ^ab^	203.77 ± 12.49 ^a^
Propanoic acid, 2-hydroxy-2-methyl-, ethyl ester	830	59	3.46 ± 0.16 ^b^	2.47 ± 0.12 ^c^	1.00 ± 0.23 ^d^	4.13 ± 0.13 ^a^
Octane	835	85	3.42 ± 0.39 ^b^	3.98 ± 0.33 ^b^	7.35 ± 1.76 ^a^	6.17 ± 0.42 ^a^
Propanoic acid	837	74	1.02 ± 0.35 ^b^	2.68 ± 0.76 ^b^	5.03 ± 1.66 ^a^	2.82 ± 0.94 ^b^
1-Pentanol	860	55	40.35 ± 0.92 ^a^	11.08 ± 0.75 ^c^	9.57 ± 0.28 ^c^	14.48 ± 0.42 ^b^
Prenol	869	71	2.54 ± 0.54 ^b^	4.53 ± 0.30 ^a^	0.89 ± 0.03 ^c^	1.30 ± 0.65 ^bc^
Hexanal	878	56	135.79 ± 5.78 ^d^	183.42 ± 3.42 ^c^	299.82 ± 25.45 ^a^	238.40 ± 2.87 ^b^
Propanoic acid, 2-methyl-	901	73	0.64 ± 0.2 ^b^	1.99 ± 0.44 ^a^	2.77 ± 0.20 ^a^	1.83 ± 0.48 ^a^
2,3-Butanediol	924	45	1.85 ± 0.24 ^c^	10.39 ± 2.17^a^	3.42 ± 0.47 ^bc^	6.02 ± 0.71 ^b^
2,3-Butanediol, [R-(R*,R*)]-	932	45	14.61 ± 7.04 ^c^	107.30 ± 15.60 ^a^	69.60 ± 2.26 ^b^	65.07 ± 2.17 ^b^
Butanoic acid	933	60	101.64 ± 5.86 ^c^	377.09 ± 7.29 ^b^	447.78 ± 58.78 ^a^	352.37 ± 10.48 ^b^
Pentane, 2,2-dimethyl-	950	57	0.40 ± 0.18	0.36 ± 0.03	0.64 ± 0.18	0.54 ± 0.03
2-Heptanone	983	58	3.35 ± 0.15 ^b^	3.45 ± 0.27 ^b^	4.16 ± 0.18 ^a^	3.88 ± 0.16 ^ab^
Heptanal	990	70	8.69 ± 0.71 ^c^	12.75 ± 1.84 ^b^	14.78 ± 1.46 ^b^	18.42 ± 0.14 ^a^
.alpha.-Phellandrene	994	93	2.69 ± 0.05 ^b^	2.78 ± 0.12 ^b^	3.62 ± 0.13 ^a^	3.82 ± 0.02 ^a^
Pentanoic acid	1021	60	2.91 ± 0.59	4.32 ± 0.77	4.31 ± 0.50	4.14 ± 1.03
Dimethyl trisulfide	1052	126	0.06 ± 0.04 ^c^	0.84 ± 0.29 ^b^	0.49 ± 0.20^b^	10.19 ± 3.59^a^
Furan, 2-pentyl-	1055	81	12.19 ± 0.30 ^d^	17.36 ± 0.80 ^c^	41.45 ± 5.14 ^a^	26.19 ± 1.01 ^b^
Benzaldehyde	1063	106	20.80 ± 1.14 ^b^	14.98 ± 1.32 ^b^	49.22 ± 2.72 ^a^	53.63 ± 6.10 ^a^
1-Octen-3-ol	1069	57	11.33 ± 0.66 ^c^	13.87 ± 0.63 ^b^	22.84 ± 1.68 ^a^	14.42 ± 0.58 ^b^
5-Hepten-2-one, 6-methyl-	1074	108	2.48 ± 0.11 ^a^	1.84 ± 0.18 ^b^	2.21 ± 0.10 ^a^	1.79 ± 0.08 ^b^
Octanal	1085	84	4.97 ± 0.32 ^c^	6.27 ± 1.45 ^bc^	8.84 ± 0.85 ^ab^	8.92 ± 0.12 ^a^
Undecane, 3,6-dimethyl-	1087	57	14.29 ± 2.23 ^b^	5.39 ± 0.27 ^c^	5.82 ± 0.64 ^c^	49.82 ± 1.31 ^a^
Dodecane, 2,6,10-trimethyl-	1098	57	11.79 ± 1.24 ^b^	4.12 ± 0.39 ^c^	3.80 ± 0.28 ^c^	39.75 ± 3.40 ^a^
Hexanoic acid	1102	60	14.99 ± 0.58 ^b^	31.96 ± 4.88 ^a^	25.57 ± 3.12 ^a^	25.40 ± 1.98 ^a^
2-Ethyl-1-hexanol	1113	57	3.00 ± 0.16 ^c^	3.71 ± 0.11 ^b^	5.23 ± 0.06 ^a^	3.55 ± 0.20 ^b^
Butane, 2,2-dimethyl-	1133	57	0.53 ± 0.01 ^d^	0.66 ± 0.02 ^c^	0.87 ± 0.12 ^b^	1.21 ± 0.03 ^a^
1-Octanol	1147	55	0.95 ± 0.25	0.72 ± 0.01	0.99 ± 0.16	0.98 ± 0.18
1-Hexen-3-one	1152	70	1.53 ± 0.17 ^b^	0.73 ± 0.03 ^c^	0.53 ± 0.11 ^c^	4.85 ± 0.47 ^a^
Nonanal	1169	56	7.84 ± 0.38	12.20 ± 4.35	12.71 ± 1.49	11.90 ± 0.34
Undecane, 4,4-dimethyl-	1174	85	1.91 ± 0.17 ^b^	0.75 ± 0.07 ^c^	0.55 ± 0.03 ^c^	4.37 ± 0.33 ^a^
Undecane, 3-methyl-	1189	57	2.08 ± 0.01 ^b^	1.53 ± 0.09 ^c^	1.17 ± 0.08 ^d^	4.50 ± 0.12 ^a^
Dodecane	1210	71	3.76 ± 0.15 ^b^	3.05 ± 0.11 ^b^	3.54 ± 0.17 ^b^	5.67 ± 0.50 ^a^
Decane, 5-ethyl-5-methyl-	1226	71	2.46 ± 0.20 ^b^	0.73 ± 0.08 ^c^	0.41 ± 0.06 ^c^	4.87 ± 0.10 ^a^
2,2,5-Trimethylhexan-4-one	1236	57	0.55 ± 0.08 ^b^	0.14 ± 0.04 ^c^	0.12 ± 0.03 ^c^	0.85 ± 0.12 ^a^
Undecane, 5-ethyl-	1241	57	1.73 ± 0.24 ^a^	0.39 ± 0.04 ^b^	0.23 ± 0.10 ^b^	2.36 ± 0.70 ^a^
Tridecane	1282	57	1.16 ± 0.06 ^c^	3.26 ± 0.03 ^b^	4.42 ± 0.61 ^a^	1.46 ± 0.17 ^c^
2,2,6,6-Tetramethylheptane	1330	57	1.43 ± 0.15 ^a^	0.56 ± 0.04 ^c^	0.53 ± 0.05 ^c^	0.99 ± 0.10 ^b^
Hexane, 3,3-dimethyl-	1348	57	1.68 ± 0.24 ^a^	1.28 ± 0.13 ^ab^	1.44 ± 0.23 ^ab^	0.88 ± 0.10 ^b^
Pentadecanal	1548	82	0.56 ± 0.06 ^c^	0.73 ± 0.06 ^c^	1.97 ± 0.09 ^a^	1.11 ± 0.04 ^b^

Values are means ± standard deviation. ^a, b, c, d^ Means in the same column followed by different lowercase letters present statistically significant difference by the Tukey test (*p < 0.05*). LRI: linear retention index calculated for DB-624 capillary column (J&W scientific: 30 m × 0.25 mm i.d., 1.4 µm film thickness) installed on a gas chromatograph equipped with a mass selective detector. *m/z*: Quantifier ion. FC1: 100% NaCl; F1: 50% NaCl + 50% KCl; F2: 50% NaCl + 50% CaCl_2_; F3: 50% NaCl + 25% KCl + 25% CaCl_2._

**Table 5:** Free fatty acids (%) of salted meat (0 day of storage).

Free Fatty Acids	Treatments			
	FC1	F1	F2	F3
C6:0	0.20 ± 0.13 ^a^	0.15 ± 0.02 ^a^	0.24 ± 0.05 ^a^	0.12 ± 0.07 ^a^
C8:0	0.14 ± 0.27 ^ab^	0.13 ± 0.01 ^b^	0.18 ± 0.31 ^a^	0.16 ± 0.12 ^ab^
C10:0	0.10 ± 0.02 ^a^	0.11 ± 0.02 ^a^	0.14 ± 0.03 ^a^	0.14 ± 0.01 ^a^
C11:0	0.11 ± 0.01 ^a^	0.13 ± 0.02 ^a^	0.18 ± 0.01 ^a^	0.19 ± 0.01 ^a^
C12:0	0.12 ± 0. 02 ^a^	0.11 ± 0.01 ^a^	0.11 ± 0.01 ^a^	0.12 ± 0.05 ^a^
C13:0	0.08 ± 0.02 ^a^	0.08 ± 0.01 ^a^	0.09 ± 0.02 ^a^	0.09 ± 0.01 ^a^
C14:0	1.12 ± 0.15 ^a^	0.97 ± 0.07 ^a^	1.22 ± 0.46 ^a^	1.25 ± 0.64 ^a^
C14:1n-5	0.21 ± 0.01 ^a^	0.16 ± 0.02 ^a^	0.15 ± 0.05 ^a^	0.19 ± 0.06 ^a^
C15:0	0.23 ± 0.02 ^a^	0.24 ± 0.03 ^a^	0.24 ± 0.02 ^a^	0.20 ± 0.06 ^a^
C15:1n-5	2.43 ± 0.64 ^a^	2.65 ± 0.53 ^a^	3.78 ± 0.16 ^a^	3.83 ± 0.27 ^a^
C16:0	23.43 ± 3.16 ^a^	22.25 ± 1.95 ^b^	22.24 ± 0.01 ^b^	24.14 ± 4.45 ^a^
C16:1n-7	1.39 ± 0.22 ^a^	1.16 ± 0.08 ^a^	1.12 ± 0.02 ^a^	1.40 ± 0.39 ^a^
C17:0	0.64 ± 0.08 ^ab^	0.74 ± 0.05 ^ab^	0.79 ± 0.08 ^a^	0.58 ± 0.16 ^b^
C18:0	22.57 ± 2.81 ^ab^	23.76 ± 2.02 ^a^	24.43 ± 0.46 ^a^	20.80 ± 2.89 ^b^
9t-C18:1	0.33 ± 0.05 ^b^	0.67 ± 0.20 ^a^	0.58 ± 0.08 ^ab^	0.28 ± 0.07 ^b^
11t-C18:1	1.24 ± 0.14 ^a^	0.98 ± 0.28 ^a^	1.07 ± 0.02 ^a^	1.36 ± 0.32 ^a^
C18:1n-9	24.19 ± 2.69 ^a^	26.76 ± 2.31 ^a^	26.09 ± 1.45 ^a^	24.85 ± 6.99 ^a^
C18:1n-7	1.42 ± 0.21 ^b^	1.68 ± 0.13 ^a^	1.66 ± 0.02 ^a^	1.37 ± 0.26 ^b^
9t,11t-C18:2	0.22 ± 0.04 ^a^	0.16 ± 0.02 ^a^	0.18 ± 0.01 ^a^	0.23 ± 0.01 ^a^
C18:2n-6	12.65 ± 1.30 ^a^	11.22 ± 0.66 ^a^	9.55 ± 0.32 ^b^	11.53 ± 1.60 ^a^
C18:3n-6	0.19 ± 0.04 ^a^	0.11 ± 0.02 ^b^	0.12 ± 0.03 ^b^	0.18 ± 0.02 ^ab^
C18:3n-3	1.23 ± 0.13 ^a^	0.61 ± 0.02 ^b^	0.60 ± 0.02 ^b^	1.15 ± 0.24 ^a^
9c,11t-C18:2 (CLA)	0.39 ± 0.05 ^a^	0.31 ± 0.02 ^ab^	0.20 ± 0.04 ^b^	0.41 ± 0.13 ^a^
C20:0	0.19 ± 0.03 ^a^	0.21 ± 0.02 ^a^	0.26 ± 0.02 ^a^	0.22 ± 0.01 ^a^
C20:1n-9	0.12 ± 0.01 ^a^	0.17 ± 0.03 ^a^	0.18 ± 0.01 ^a^	0.18 ± 0.06 ^a^
C20:2n-6	0.12 ± 0.01 ^a^	0.11 ± 0.02 ^a^	0.13 ± 0.01 ^a^	0.14 ± 0.02 ^a^
C20:3n-6	0.76 ± 0.08 ^a^	0.77 ± 0.06 ^a^	0.76 ± 0.04 ^a^	0.73 ± 0.09 ^a^
C20:4n-6	2.88 ± 0.39 ^a^	2.70 ± 0.12 ^a^	2.63 ± 0.11 ^a^	2.74 ± 0.22 ^a^
C20:5n-3	0.85 ± 0.08 ^ab^	0.60 ± 0.01 ^c^	0.72 ± 0.03 ^bc^	0.96 ± 0.09 ^a^
C22:6n-3	0.46 ± 0.10 ^a^	0.31 ± 0.05 ^a^	0.29 ± 0.11 ^a^	0.45 ± 0.18 ^a^

Values are means ± standard deviation. ^a, b, c^ Means in the same row followed by different lowercase letters present statistically significant difference by the Tukey test (*p* < 0.05). RT: retention time. FC1: 100% NaCl; F1: 50% NaCl + 50% KCl; F2: 50% NaCl + 50% CaCl_2_; F3: 50% NaCl + 25% KCl + 25% CaCl_2._
